# Medinal in Mental Disease

**Published:** 1909-09

**Authors:** 


### MEDINAL IN MENTAL DISEASE.

Peretti, Akademischer Vortrags und Demonstrationsabend in Duesseldorf; session of April 27, 1909 (*Medizinische Klinik,* July 4, 1909) reported the experiences with medinal in the Grafenberger Institute. The remedy was used in forty-two cases of mental disease of various classes —altogether about five hundred doses of generally 0.55 gm. (8 grains) to 30 gm. (1 oz.) aq. menth. The result was very similar to that from veronal, only the soporific effect was seen earlier; a rapid accustomance to the remedy, cumulative effect or undesirable by-effects were never observed. The greater solubility of medinal in cold water offers the advantage that not only can it be more conveniently given by mouth and is absorbed quicker, but also that in cases where the stomach should be spared it can be administered rectally and subcutaneously. But the subcutaneous injections, even of 1 per cent. solution (tried in four cases), are not painless:

Medinal acts best in mild conditions of depression and simple insomnia of neurasthenic character; in severe depressions the nights were passed somewhat better than without it. In restlessness due to organic causes (senile dementia, paresis) the effect was uncertain; senile women reacted better than senile men; in paralytics it often failed. In conditions of excitement of the insane, medinal can not supplant the other subcutaneous medicaments such as morphium and scopolamin, so that it is not the subcutaneous hypnotic for these cases which Ziehen has asked for.

